# Vertically Aligned Nanocrystalline Graphite Nanowalls for Flexible Electrodes as Electrochemical Sensors for Anthracene Detection

**DOI:** 10.3390/s24227194

**Published:** 2024-11-10

**Authors:** Marius C. Stoian, Octavian G. Simionescu, Cosmin Romanitan, Gabriel Craciun, Cristina Pachiu, Antonio Radoi

**Affiliations:** National Institute for Research and Development in Microtechnologies—IMT Bucharest, 126A Erou Iancu Nicolae Street, 077190 Voluntari, Romania; octavian.simionescu@imt.ro (O.G.S.); cosmin.romanitan@imt.ro (C.R.); cristina.pachiu@imt.ro (C.P.)

**Keywords:** nanocrystalline graphite, graphite nanowalls, flexible substrate, electrochemical sensor, anthracene detection

## Abstract

Plasma-enhanced chemical vapor deposition (PECVD) was used to obtain several graphite nanowall (GNW)-type films at different deposition times on silicon and copper to achieve various thicknesses of carbonic films for the development of electrochemical sensors for the detection of anthracene. The PECVD growth time varied from 15 min to 30 min to 45 min, while scanning electron microscopy (SEM) confirmed the changes in the thickness of the GNW films, revealing a continuous increase in the series. X-ray diffraction (XRD) analysis revealed that the crystallinity of the GNW film samples increased with increasing crystallite size and decreasing dislocation density as the deposition time increased. Electrochemical characterization of the GNW-based electrodes indicated that the electroactive area and heterogeneous electron transfer rate constant were greater for the GNW 45 min film in the carbonic material series. We present the transfer of GNW films on flexible polyethylene substrates for achieving flexible electrochemical sensors for further use in anthracene determination. The flexible GNW-based electrodes were investigated using differential pulse voltammetry (DPV) in the presence of anthracene. The results showed that the highest sensitivity in anthracene detection was provided by the sensor with the GNW film obtained after 45 min of PECVD growth. The optimization of the GNW film thickness for the development of flexible electrochemical sensors on polyethylene substrates represents a successful approach for enhancing the electrochemical performance of carbonic materials.

## 1. Introduction

Anthracene is one of the most common polycyclic aromatic hydrocarbons (PAHs) and is an important environmental pollutant that has been shown to have carcinogenic and mutagenic effects on humans [1]. It results from numerous industrial processes and is easily transported over long distances, leading to high concentrations in different natural resources such as air, water, soil, and sediments, which ultimately leads to its high presence in water and food for human consumption [2,3]. Taking into account the severe concerns related to pollution, the European Parliament and Council encouraged new legislation toward monitoring and limiting the spread of contaminants in the environment. With more focus on water pollution, the European Water Framework Directives (WFD 2000/60/CE and 2013/39/UE) were established, which contain 45 priority chemicals that must be monitored in lakes, rivers, and seas, among which are a class of PAHs [4,5]. In the case of anthracene, the standard limits for detection in water were set by the EU Agency and US EPA at 0.1 μg/L and 0.034 μg/L, respectively [5,6].

Previous papers have touched upon the need for reliable detection of anthracene and other PAHs with the possibility of ease of use and portability for on-site analysis to monitor and control the toxicity in the environment [7,8,9,10]. In the context of PAH detection, the electrochemical approach represents an attractive alternative to conventional analysis techniques, including high-performance liquid chromatography (HPLC), gas chromatography in combination with UV–Vis spectroscopy, mass spectrometry (MS), or fluorescence detection. These methods are the most sensitive and are widely used in environmental analysis [11,12]. However, they require expensive equipment, which is difficult to transport for on-site determination, and sample processing before analysis. Electrochemical methods can provide similar results with the use of inexpensive reagents and equipment, which are portable for on-site measurements, have a shorter analysis time, and have no need for highly skilled personnel [7].

In addition to electrochemical sensors for PAH detection in the environment developed in recent years, which use different nanomaterials (metal nanoparticles, polyanilines, graphene oxide, or polypyrrole) or conductive substrates (glassy carbon electrode, gold, indium-tin oxide, screen-printed carbon electrode, or silicon) [6,7,8,13,14], carbon-based films have also been widely used for developing electrode materials suitable for the detection of pollutants and toxic compounds [15,16,17]. Owing to its excellent electrical and mechanical properties, carbon-based materials, such as graphene, carbon nanotubes, graphitized carbon, etc., have also been found in multiple devices such as photodetectors, bandwidth absorbers, and supercapacitors for sensing and energy storage applications [18,19,20,21,22]. One of our authors’ previous papers showcases the beneficial use of nitrogen-doped bulk nanocrystalline graphite (N-doped bulk NCG) for the electrochemical detection of anthracene. These carbon materials of multiple low-range ordered sp^2^-conjugated domains surrounded by sp^3^ amorphous carbon have been proven to provide an interesting platform for the electrochemical detection of anthracene, as nitrogen doping of such films grown on a silicon substrate improved their sensing capabilities without requiring preactivation of the electrode [9].

Flexible polymeric-based sensors offer a cost-effective alternative for achieving more robust electrochemical sensors for the determination of pollutants compared to the usual development of sensors on rigid substrates. In addition, using easily biodegradable polymers provides both an environmentally and economically advantageous approach, especially compared to the high costs associated with highly doped crystalline silicon [23,24]. In this regard, a more recent paper also explored flexible electrochemical sensors based on the NCG material class [10]. In the respective work, the use of a polyethylene (PE) layer as a flexible substrate for obtaining electrochemical sensors based on both N-doped bulk NCG and graphene/graphite nanowall (GNW) films for the determination of anthracene was explored. The GNW thin film—which is part of the NCG material class as it shares a similar internal structure to bulk NCG but has a distinct and specific morphology—demonstrated good compatibility with the PE layer. Although the N-doped bulk NCG film displayed a greater sensing performance for anthracene when tested on Si substrates, the flexible GNW-based electrode, which had a higher C sp^2^ hybridization rate, showed a higher sensitivity and lower detection limit in the anthracene electrochemical determinations when compared to its flexible N-NCG-based electrode counterpart [10].

Therefore, in light of these findings, the present work aims to optimize the GNW film thickness and surface distribution for developing enhanced flexible electrochemical sensors for anthracene detection. The optimization of the GNW film thickness on the PE substrate should afford enhanced sensing performance in anthracene determination, as the morphologic and structural features of the carbonic films translate into changes in electrochemical properties with a significant impact on the sensitivity of the sensors.

## 2. Experimental Section

### 2.1. Materials and Reagents

The silicon wafers (B-doped, 1–5 mΩ cm resistivity, (100) orientation, 4-inch diameter and 525 µm thickness) used for the PECVD growth were purchased from SIEGERT WAFER GmbH (Aachen, Germany). The Cu foils (35 μm thick, 99.95% purity) were obtained from Graphene Platform Corporation (Tokyo, Japan) and used for the growth process. Potassium hexacyanoferrate (III) (K_3_[Fe(CN)_6_]), potassium chloride (KCl), iron(III) chloride (FeCl_3_), phosphate-buffered saline (PBS), lithium perchlorate (LiClO_4_), anthracene (ANTR), phenanthrene (PHEN), naphthalene (NAPHT) and fluoranthene (FLUOR) were purchased from Sigma-Aldrich (St. Louis, MO, USA). The purity of these materials was of analytical grade (99.0–100.5% purity), and they were used as received without further purification. Acetonitrile (ACN), HPLC gradient grade, ≥99.9% purity, was purchased from Riedel-de Haen, Honeywell (Seelze, Germany). The polyethylene films (Meltonix, 60 µm) were provided by Solaronix (Aubonne, Switzerland) and used in the transfer process of the carbonic films. During most of the different processing and measurement steps, deionized (DI) water was used.

### 2.2. Carbonic Material Growth Process and Transfer Process onto PE Substrate

The GNW thin films were grown on both Si substrates and Cu foils within a Nanofab 1000 PECVD reactor (Oxford Instruments, Abingdon, UK) at a substrate temperature of ~750 °C, with similar process steps and parameters as those used in [10]. Prior to deposition, the Si substrates were thoroughly cleaned in isopropanol and acetone, while the Cu foils were cleaned in nitric acid, DI water, and isopropanol. All substrates were then dried under a nitrogen flow. To investigate thin films of different nanowall heights and surface distribution, the time of the deposition step from [10]—which takes place at 300 W RF power, 190:10 sccm Ar:CH_4_ flow rate, and 300 mTorr pressure—was varied from 15 min to 30 min and ultimately to 45 min. The films grown on silicon were further characterized, whereas the GNW films grown on copper were subsequently transferred to flexible PE substrates cut to specific sizes and tested for anthracene determination.

The transfer process, which is extensively presented in [10] and showcased in Figure S1, consists of the following: applying 20 × 15 mm PE layers on both sides of the GNW/Cu assembly; bonding the PE layers to the GNW/Cu assembly by means of a thermocompression process at 100 °C for 30 min; selectively etching the Cu foil through the backside with 1 M FeCl_3_ solution [25] through a predetermined open area of the PE foil on the Cu face, as illustrated in Figure S2. The electrodes obtained on PE substrates through this process presented flexible and bendable properties, as seen in Figure S3, where the electrode in a bent position was able to return to its original shape.

The transfer process did not include additional binders or other reagents for the binding step, which considered only the adhesion properties of the polyethylene substrate. This approach has one major advantage over other techniques for achieving flexible electrochemical sensors via conductive inks, direct writing/ball pen writing, and screen printing, which use binders for adhesion [26,27].

### 2.3. Material Characterization and Electrochemical Investigation

Morphological and structural investigations of the carbon-based materials were performed with a field emission scanning electron microscope (FE-SEM) FEI-NOVA NanoSEM 630 (FEI Company, Hillsboro, OR, USA), at different accelerating voltages (10 and 15 kV) to acquire cross-section and top-view micrographs of the GNW film samples. The cross-section SEM micrographs were used to evaluate the layer thickness of the PECVD-grown GNW films. The width of the nanowalls in the carbon materials was analyzed from the top-view SEM micrographs using the open source software ImageJ version 1.54j 2024 to determine the mean value and the range distribution [28].

X-ray diffraction (XRD) of the GNW films was performed with a diffraction system Rigaku SmartLab with a 9 kW rotating anode (Osaka, Japan) and CuK_α1_ radiation (λ = 1.5406 Å) at 45 kV and 100 mA. The incidence angle of the source in the grazing incidence X-ray diffraction (GI-XRD) measurements was set to 0.5°, while the detector scanned at a 2*θ* angle from 10° to 90°. The incidence slit was set to 0.1 mm, and the receiving slits were set to 20 mm.

Raman spectroscopy measurements on the GNW films were carried out by a high-resolution Scanning Near-Field Optical Microscope fitted with the Raman Module Witec Alpha 300S (Witec, Ulm, Germany), using a laser with a 532 nm wavelength (laser excitation energy of 2.33 eV).

The electrochemical characterization of the GNW-based electrodes was carried out with an Autolab electrochemical system (model PGSTAT 302 N) equipped with FRA 32 M, SCAN 250, and apparent diffusion coefficient (ADC) 10 M modules (Metrohm, Utrecht, the Netherlands). The GNW-based electrodes were investigated in a Teflon flat cell in a three-electrode configuration: for aqueous media (i.e., 10 mM PBS + 0.1 M KCl), the reference electrode used was a Ag/AgCl electrode, while for measurements in organic/aqueous solutions (ACN/H_2_O, 80/20 *v*/*v* + 0.1 M LiClO_4_), the reference electrode was a Ag/AgNO_3_ electrode; both electrodes were provided by Redox.me (Krakow, Poland). The GNW-based electrodes were considered the working electrodes, and a platinum wire was considered the auxiliary electrode. Cyclic voltammograms (CVs) were recorded within the −0.2–0.8 V potential window at different scan rates using a solution of 2 mM K_3_[Fe(CN)_6_] with 10 mM phosphate-buffered saline (PBS) as the supporting electrolyte and 0.1 M KCl. Electrochemical impedance spectroscopy (EIS) spectra were acquired within the 0.1 Hz–100 kHz frequency range in a solution containing 2 mM K_3_[Fe(CN)_6_] with 10 mM PBS and 0.1 M KCl as the supporting electrolyte. EIS data were acquired at the equilibrium potential (0.24 V DC) and 10 mV AC. CVs were generated in the presence of polycyclic aromatic hydrocarbons (ANTR and PHEN) in mixed organic/water media (ACN/water, 80/20 *v*/*v* + 0.1 M LiClO_4_) and acquired in the 0–1.4 V potential window. The GNW-based electrodes on PE were tested through differential pulse voltammetry (DPV) measurements in the 1 μM–2 mM anthracene concentration range and 0–1.2 V potential window to evaluate the sensitivity of the GNW films obtained at different deposition times.

## 3. Results and Discussion

### 3.1. Morphological and Structural Characterization of the GNW Films

**Figure 1:**
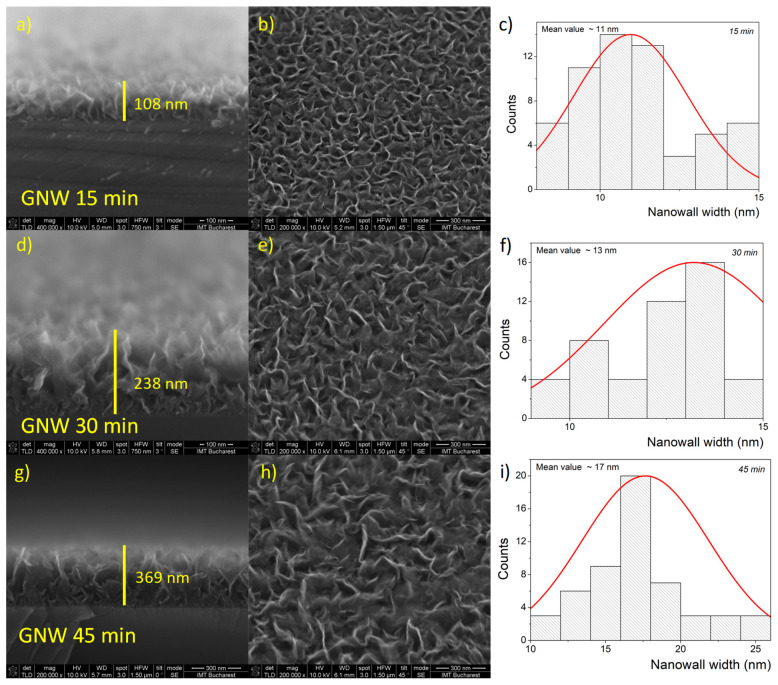
Cross-sectional (**a**,**d**,**g**) and (**b**,**e**,**h**) top-view SEM micrographs of GNW films grown for different deposition durations on a Si substrate with the corresponding distribution across the nanowall width (**c**,**f**,**i**).

The growth of the GNW films on silicon substrates was evidenced through SEM, which indicates a different nanowall height for the utilized deposition times as well as several changes in surface morphology (Figure 1). An increase in the layer thickness for the GNW films grown on Si, from 108 nm to 238 nm and finally to 369 nm was clearly observed for deposition times of 15 min, 30 min, and 45 min, respectively, as illustrated in the cross-sectional SEM micrographs (Figure 1a,d,g). The top-view SEM micrographs revealed clear thin graphite nanowalls, which originated from several nucleation points and extended in different directions (Figure 1b,e,h). Although the PECVD process allows additional nucleation on existing nanowalls and further ramification, the increasing growth time appears to favor the development of existing nanowalls [29], which develop increasingly larger and less numerous, indicating a more crystalline and compact graphite material layer. Moreover, the width of the nanowalls tends to increase with a higher growth time, from ~11 nm for GNWs obtained at 15 min to approximately 17 nm for GNWs obtained at 45 min (Figure 1c,f,i).

**Figure 2:**
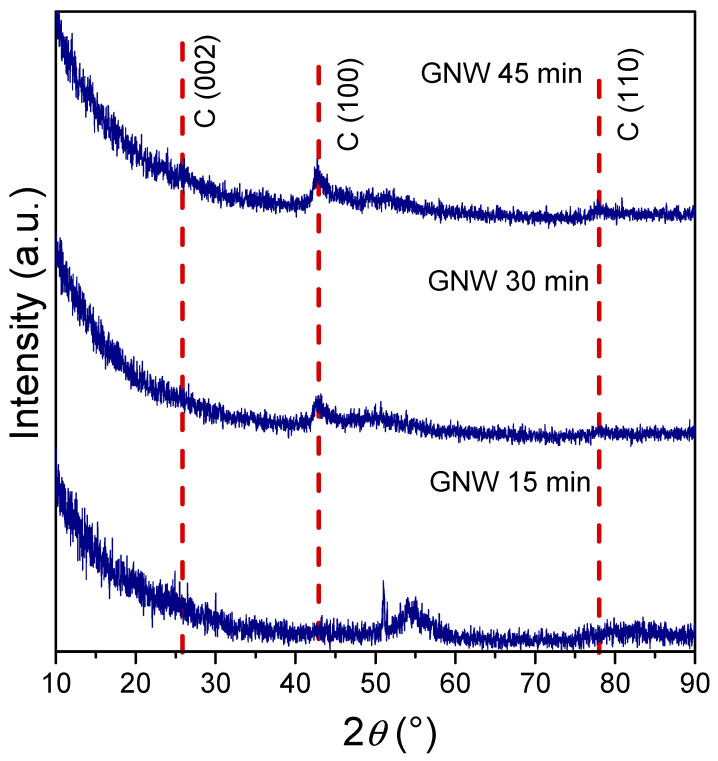
XRD patterns of GNW films obtained at different growth times.

The analysis of the GNW films via X-ray diffraction as a function of growth time revealed different microstructural features for each sample (Figure 2). After 15 min, the XRD pattern displays weak diffraction features corresponding to a carbon phase, which are barely discernable at ~42.9°. Only a diffraction peak attributed to the Si substrate can be observed at ~53°. However, at longer growth times of 30 min, the XRD pattern displays a diffraction feature occurring at 2*θ* = 42.9°, assigned to the (100) reflection of graphitic carbon, and a weak feature at 2*θ* = 78° assigned to the (110) reflection. Moreover, by further increasing the growth time to 45 min, the diffraction features assigned to C (002), C (100), and C (110) reflections become relatively more prominent as they evolve at 2*θ* = 25.9°, 42.9°, and 78°, respectively. Peak indexing was performed using the International Centre for Diffraction Data (ICDD) database with card no. 01-0640.

The mean crystallite size for the investigated samples was analyzed by the Scherrer equation using the C (100) reflection. The mean crystallite size, D, is related to the full width at half maximum (FWHM), β, of the diffraction peaks; thus [30],

(1) $$D=\frac{k\lambda }{\beta \cos\theta },$$

where k is the shape factor of the crystallites, taken as 0.93, *λ* is the X-ray wavelength (0.15406 nm), and θ is the angular position of the diffraction peak.

An increase in the mean crystallite size with longer growth times was observed, from ~3.5 nm at 15 min to ~5 nm at 30 min to ~10 nm at 45 min. These results are also supported by the top-view SEM micrographs, which showed a gradual increase in the nanowall width with an increasing growth time from ~11 nm to ~17 nm. Both SEM and XRD clearly demonstrated that the growth time enhances the crystal quality of the carbonic materials.

The dislocation density (δ) in the GNW films, which is indicative of the number of defects, can be estimated using the following formula: δ = 1/*D*^2^, where *D* is the crystallite size of the graphite phase [31]. The dislocation densities obtained for the graphite phase in the GNW film series decreased with the growth time from 8.11 × 10^12^ cm^−2^ to 3.42 × 10^12^ cm^−2^ to 4.93 × 10^11^ cm^−2^ for deposition times of 15 min, 30 min and 45 min, respectively. Usually, a larger crystallite size is ascribed to a lower dislocation density in the material [9]. Thus, for the sample obtained after the longest growth time, a lower dislocation density is indicative of greater crystallinity in the carbonic film, providing enhanced conductivity properties, which are important for electrochemical measurements in sensing applications.

**Figure 3:**
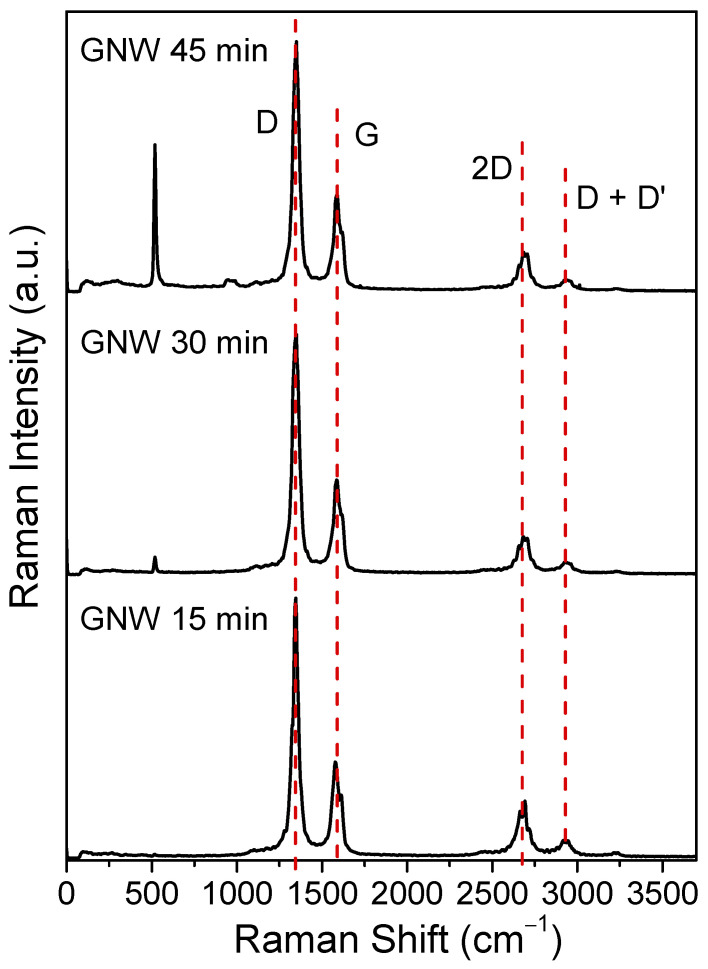
The Raman spectra of the GNW films obtained at different growth times.

The Raman spectra of the GNW films obtained at different growth times showed similar profiles with the specific bands, corresponding to the NCG class of materials, at around 1348, 1580, 2698, and 2931 cm^−1^ for the D, G, 2D, and D + D’ bands, respectively [32,33,34] (Figure 3).

The G band, which is related to sp^2^-bonded carbon atoms, presented its peak close to the ideal graphitic value of 1580 cm^−1^ in the case of the GNW 15 min film (1579 cm^−1^); however, at longer growth times, a blue shift was noticed as the G peak band reached 1588 cm^−1^ (Table S1). The blue shift is indicative of more sp^2^ discontinuities and disorder in the carbonic structure. The presence of the D, 2D, and D + D’ bands, which are related to defects and inter-layer relations, was evidenced with comparable intensities for all the GNW films in the series, revealing similar defect densities in the samples [32,33,34]. *I_D_*/*I_G_* intensity ratios were calculated, obtaining close values of 2.68, 2.44, and 2.50 for the GNW film at 15 min, 30 min, and 45 min growth times, respectively. Based on these intensity ratios, an average crystallite size can be estimated, in accordance with Equation (2) [35]:

(2) $$L_{a}(nm)={(2.4\times 10^{-10})\lambda_{l}^{4}\left(\frac{I_{D}}{I_{G}}\right)}^{-1},$$

where $L_{a}$ is the lateral size of the average crystallite (nm) and $\lambda_{l}$ is the laser wavelength in nm.

The values for the crystallite size resulted at around 7 nm for all the samples, taking into account the intensity ratios, which varied irrespective of the PECVD growth time.

### 3.2. Electrochemical Characterization of the Carbonic Films

The electron transfer properties of transducer materials can be evaluated via cyclic voltammetry (CV) in the presence of a redox mediator, which can be oxidized and reduced at the electrode surface. By comparing the position and separation of the observed redox peaks, details regarding the electrochemical activity are revealed, which describe the differences found in the tested materials. Generally, a small peak separation is indicative of high electron transfer properties, achieving values close to the Nernstian constant (59 mV) for reversible redox processes at the electrode surface.

**Figure 4:**
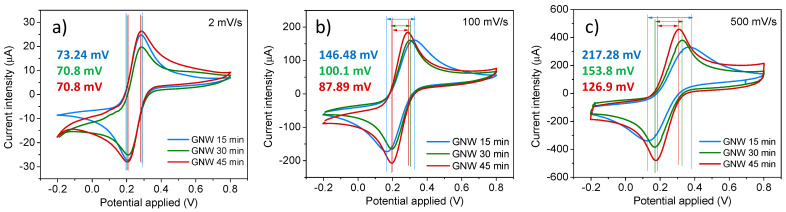
Cyclic voltammetry at (**a**) 2 mV/s, (**b**) 100 mV/s, and (**c**) 500 mV/s for the GNW films grown on Si in the presence of 2 mM K_3_[Fe(CN)_6_] dissolved in 10 mM PBS containing 0.1 M KCl.

The GNW-based electrodes on hard silicon substrates were analyzed via CV at different scanning rates (2–500 mV/s) in a solution containing 2 mM K_3_[Fe(CN)_6_] with 10 mM PBS and 0.1 M KCl. The voltammograms of the GNW films recorded at 2, 100, and 500 mV/s show well-defined peaks, which can be ascribed to the quasi–reversible redox reaction of the ferricyanide ion couple, with peak separations higher than 70 mV (Figure 4). The GNW 45 min sample exhibited the lowest peak separations at 70.8, 87.89, and 126.9 mV at all the considered scanning rates of 2, 100, and 500 mV/s, respectively, followed by the GNW 30 min (70.8, 100.1, and 153.8 mV) and GNW 15 min samples (73.24, 146.48, and 217.28 mV), indicating greater electron transfer properties for the GNW sample grown with the longest deposition step. Moreover, the GNW 45 film exhibited the highest peak current intensity within the carbonic-type material series.

**Figure 5:**
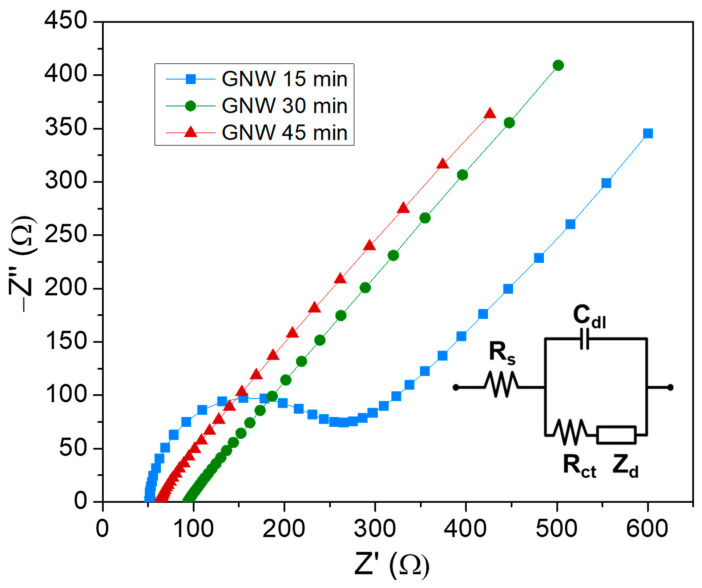
Nyquist plots of the GNW films grown on Si in the presence of 2 mM K_3_[Fe(CN)_6_] dissolved in 10 mM PBS containing 0.1 M KCl (inset the equivalent circuit; R_s_—solution resistance, C_dl_—double layer capacitance, R_ct_—charge transfer resistance, and Z_d_—diffusion impedance).

The resistivity of the carbonic materials during charge transfer can be evaluated through Nyquist plots obtained in the presence of ferricyanide at the equilibrium redox potential. Figure 5 shows the different conductive behaviors of the PECVD-grown GNW films, which have distinct profiles. For the GNW 15 min film, a semicircle is defined at high frequencies, which corresponds to grain boundary conduction, resulting in a charge transfer resistance (*R*_ct_) of approximately 264 Ω. On the other hand, the Nyquist profiles of the other films do not exhibit these features; instead, they exhibit a continuous diffusion of ions in an all-frequency range. Moreover, the charge transfer resistance is lower, with values of ~95 Ω for the GNW 30 min film and ~65 Ω for the GNW 45 min film. These results indicate increased conductivity for the GNW 45 min film, as also inferred from the XRD structural analysis, which revealed increased crystallinity for longer deposition times, such as in the case of the GNW 45 min film.

**Figure 6:**
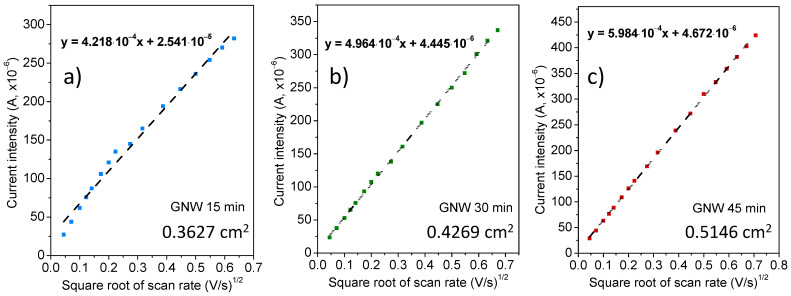
Plots of the peak current intensity versus the square root of the scan rate for the GNW/Si electrode series obtained by PECVD growth times of 15 min (**a**), 30 min (**b**), and 45 min (**c**).

The electroactive area of the GNW-based electrodes on Si can be determined from CV measurements at different scanning rates in the presence of ferricyanide by plotting the peak current intensity as a function of the square root of the scan rate (Figure 6). The Randles–Ševčík equation describes the dependence between the peak current intensity and the square root of the scan rate, as follows [36]:

(3) $$I_{p}=2.69\times 10^{5}n^{3/2}A\sqrt{D}C\sqrt{v}K\left(\Lambda ,\alpha \right),$$

where $I_{p}$ is the forward peak current, v is the scan rate, A is the electroactive area of the electrode, D is the diffusion coefficient of ferricyanide (7.3 × 10^−6^ cm^2^ s^−1^), C is the concentration of potassium ferricyanide in mol cm^−3^, n represents the number of electrons involved in the reaction (n=1), and K(Λ,α) is a modified dimensionless parameter for quasi–reversible reactions.

The experimental data of the peak currents versus the square root of the scan rate were fitted with a linear equation, providing different slopes for the GNW films obtained at different deposition times. Considering Equation (3) and the values of the slopes from the linear plots, the electroactive areas of the three GNW films can be calculated. A higher slope results in an increased electroactive area, as in the case of the GNW 45 min film, with an area of 0.5146 cm^2^, whereas the slopes of the GNW 15 min and GNW 30 min films are lower, with values of 0.3627 and 0.4269 cm^2^, respectively. The increase in the electroactive area for the GNW-based electrodes can be mainly ascribed to the increase in carbonic film thickness (i.e., nanowall height) and the improvement in material crystallinity (with effect on the conductivity) with deposition time.

Additionally, the heterogeneous electron transfer (HET) rate constant (*k*^o^) can be calculated with the data obtained from the CV measurements in the presence of ferricyanide at different scan rates to evaluate the redox ability of the GNW-based electrodes by Nicholson’s method, which is specific to quasi–reversible electrochemical reactions [36,37]:

(4) $$k^{o}=\Psi \sqrt{\pi DnvF/RT},$$

where D is the diffusion coefficient of ferricyanide (7.3 × 10^−6^ cm^2^ s^−1^), n is the number of electrons transferred (n=1), v is the scan rate, F is the Faraday constant (F = 96,485 C mol^−1^), R is the ideal gas constant (R = 8.314 J mol^−1^ K^−1^), T represents the temperature in Kelvin (T = 298 K), and Ψ is a dimensionless kinetic parameter defined as follows: $\Psi =(-0.6288+0.0021\Delta E_{p})/(1-0.017\Delta E_{p})$, where $\Delta E_{p}$ represents the peak-to-peak separation ($\Delta E_{p}=E_{pa}-E_{pc}$).

Applying Equation (4) for each scan rate used in the CV measurements of the GNW-based electrodes resulted in the following average heterogeneous electron transfer rate constants: 2.86 × 10^−3^ cm s^−1^ for the GNW 15 min film, 5.24 × 10^−3^ cm s^−1^ for the GNW 30 min film, and 7.16 × 10^−3^ cm s^−1^ for the GNW 45 min film. As the value of the peak separation found in the carbonic series starts to decrease with increasing PECVD growth time (Table S2), these HET rate constants indicate that the redox transfer capacity of the GNW 45 min film is greater than that of the other films, demonstrating its higher potential for polycyclic aromatic hydrocarbon detection.

### 3.3. Electrochemical Determination of Anthracene on GNW-Based Flexible Electrodes

**Figure 7:**
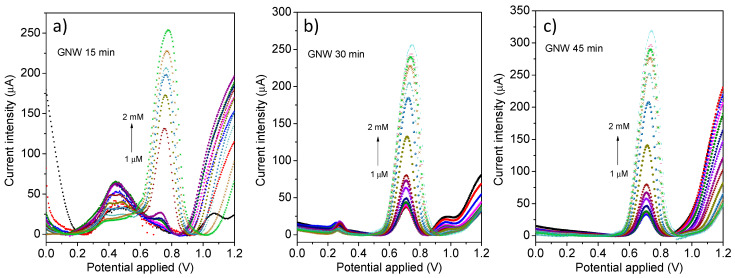
DPV measurements of the flexible GNW-based electrodes—15 min (**a**), 30 min (**b**), and 45 min (**c**)—in the presence of different anthracene concentrations (1 µM–2 mM) in an acetonitrile/water mixture (ACN/H_2_O, 80/20 *v*/*v*) with 0.1 M LiClO_4_.

The GNW films transferred on PE substrates showed good electrocatalytic properties toward anthracene oxidation, as evidenced by the CV profiles obtained in the presence of 1 mM anthracene dissolved in ACN/water (*v*/*v* = 80/20) containing 0.1 M LiClO_4_ as the supporting electrolyte, where the hydrocarbon was oxidized at a potential of 0.89 V (Figure S4). Furthermore, DPV was used as the electroanalytical method for a more sensitive determination of the current intensity variation with the addition of anthracene. The GNW-based flexible electrodes were tested via DPV at different concentrations of anthracene in the solution (1 μM–2 mM). A continuous increase in the current intensity was observed in the 0.7–0.8 V potential region, ascribed to the oxidation of anthracene, for all GNW films (Figure 7).

**Figure 8:**
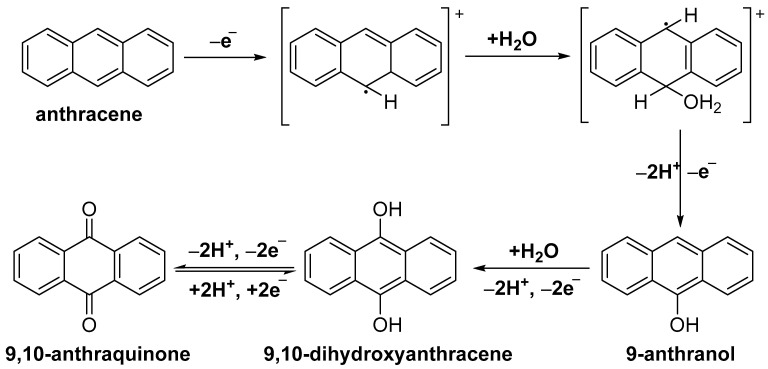
Proposed mechanism for the electrochemical oxidation of anthracene in an acetonitrile/water mixture.

A decrease in the potential value of the anthracene oxidation peak and an increase in its current intensity can be noticed with the increasing PECVD growth time, indicating the GNW 45 min film as more sensitive. A mechanism of the electrochemical oxidation of anthracene at the carbonic electrode surface in an acetonitrile/water solvent mixture is proposed and depicted in Figure 8. The first step of the oxidation consists in the PAH molecule losing an electron to the electrode via a direct electron transfer; this process generates π-delocalized cationic radicals that can react with other nucleophile agents, resulting in an irreversible oxidation process [38]. Furthermore, water molecules react with the carbonic radicals, favoring the formation of hydroxy compounds, which can be oxidized in the final step into 9,10-anthraquinone [10,38].

**Figure 9:**
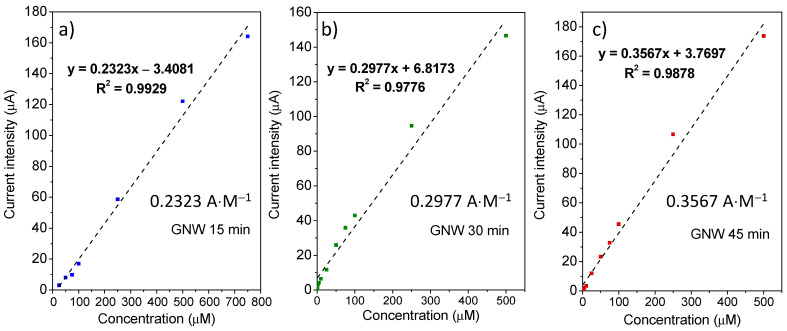
Calibration curves for the flexible GNW-based electrodes for anthracene determination: GNW films obtained after PECVD growth times of 15 min (**a**), 30 min (**b**), and 45 min (**c**).

Several calibration curves, with high correlation coefficients, were obtained for the flexible GNW-based electrodes by plotting the current intensities as a function of the anthracene concentration in the solution (Figure 9). The higher values of the slope of the calibration curves of the films grown for a longer duration are indicative of increased sensitivity toward the tested analyte. Thus, there was a clear variation in the sensitivity towards anthracene of the flexible GNW-based electrode series, as the slopes increased with the deposition time of the carbonic materials. For the GNW 15 min sample, a slope of 0.2323 A/M was obtained in the linear range domain of 25–750 μM, whereas the sensitivity started to increase for the samples with longer growth times, as the GNW 30 min and 45 min films presented sensitivities of 0.2977 and 0.3567 A/M, respectively, in the linear range of 1–500 μM. Moreover, the detection limits (LOD) of the GNW-based electrode series were 26.7, 12.9, and 9.4 μM for the GNW 15 min, 30 min, and 45 min films, respectively.

These results demonstrate the capacity of the flexible GNW-based electrodes for the detection of polycyclic aromatic hydrocarbons, specifically anthracene. Moreover, the sensing performance is enhanced by optimizing the carbonic layer thickness, as the highest sensitivity is achieved for the flexible electrochemical sensor containing the GNW film grown for 45 min. These findings can be attributed to the higher crystallinity of the carbonic material, greater electroactive area, and greater heterogeneous electron transfer rate constant for the GNW 45 min film. The results obtained for the flexible GNW 45 min/PE electrode are comparable to those obtained for other electrodes used in the electrochemical determination of anthracene, as presented in Table S3. Multiple reported electrodes used in the electrochemical detection studies on anthracene required high potential values (≥0.96 V) for the anthracene electro-oxidation in comparison to the GNW-based electrodes that operate at lower potentials (0.7–0.8 V). At the same time, they deliver narrower and limited working linear ranges for the determination of anthracene. The GNW 45 min/PE electrode also showed a significantly higher sensitivity and linear working range in the anthracene determination when compared to the previous results of GNW/PE obtained after 60 min of PECVD growth time, although with a slightly higher detection limit [10]. It seems that higher growth times led to GNW films with a larger thickness, which present lower sensitivity in anthracene detection, owing to a possible agglomeration of the nanowalls that inhibits the analyte diffusion. Taking these facts into account, the optimization of the GNW film thickness was required to obtain more sensitive electrochemical sensors.

**Figure 10:**
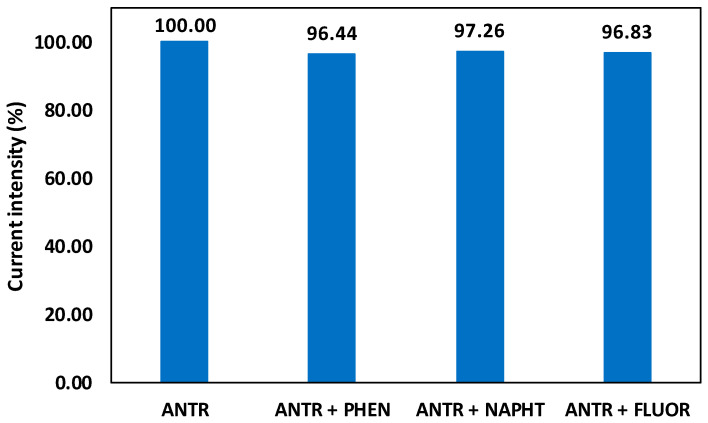
Interference tests for the GNW 45 min/PE flexible electrode in the presence of anthracene and other PAH-type compounds (phenanthrene—PHEN, naphthalene—NAPHT, and fluoranthene—FLUOR).

Additional DPV measurements were performed using the GNW 45 min/PE electrode for the determination of anthracene content in the presence of possible interferents that can appear together with the target analyte, such as other polycyclic aromatic hydrocarbons (phenanthrene, naphthalene, or fluoranthene). The current intensities of the GNW 45 min/PE electrode obtained in the presence of 500 μM of only anthracene were compared to those obtained for solutions of 500 μM anthracene containing interferent species at 300 μM (Figure 10). The small current changes in the presence of other interferents demonstrated the high selectivity of the flexible GNW-based electrode toward anthracene compared to the other polycyclic aromatic hydrocarbons.

The reproducibility of the GNW 45 min/PE electrode was evaluated through five consecutive DPV measurements on the same electrode (Figure S5). Due to the irreversible nature of the anthracene electro-oxidation process, the oxidized by-products are strongly chemosorbed on the carbon surface; thus, the current intensities indicate a decrease after each measurement, achieving around 70% of the initial intensity value after five tests. Consequently, this type of electrode is more suitable for single-time use. Further electrochemical measurements on the GNW 45 min/PE electrode were performed with real samples (tap water), which were spiked with different anthracene concentrations (100–350 μM), to evaluate the accuracy of the sensor. Table 1 shows good range of percentage recovery values (89.2% to 96.4%) for the GNW-based sensor, suggesting potential for practical applications such as detection in relevant water environments.

The electrochemical measurements indicate the ability of the GNW film to provide a sensitive and selective platform for anthracene detection, in the form of a flexible electrochemical sensor based on polyethylene, as a more friendly environment and economical choice of substrate, and an active GNW material with an optimum thickness.

## 4. Conclusions

PECVD growth was employed to obtain several GNW-type films with different deposition times on silicon and copper to achieve various thicknesses of carbonic films for the development of electrochemical sensors for the detection of anthracene. SEM images confirmed the changes in the thickness of the GNW films when the growth time was varied from 15 min to 30 min to 45 min, revealing a continuous increase in both nanowall height and thickness. XRD analysis revealed an increase in crystallinity for the GNW film samples, with larger crystallite sizes and lower dislocation density as the deposition time increased. Electrochemical characterization of the GNW-based electrodes indicated a greater available electroactive area and heterogeneous electron transfer rate constant for the GNW 45 min film in the carbonic material series. These GNW films were transferred onto flexible polyethylene substrates to achieve flexible electrodes for further use in anthracene determination. The results showed that the highest sensitivity in anthracene detection was provided by the sensor with the GNW film obtained after 45 min of PECVD growth. The flexible GNW-based sensors provided a reliable platform for anthracene detection and the optimization of the GNW film growth process proved to be a successful approach for enhancing the electrochemical performance of the carbonic materials.

## Figures and Tables

**Table 1:** Recovery values of ANTR in spiked real samples obtained using the GNW-based sensor.

Samples	ANTR Spiked (μM)	ANTR Found (μM)	Recovery (%)
Tap water	100	102.6	96.4
	200	196.4	93.6
	300	286.2	92.8
	350	337.8	89.2

## Data Availability

Data are contained within this article and Supplementary Materials.

## Supplementary Materials

The following supporting information can be downloaded at https://www.mdpi.com/article/10.3390/s24227194/s1, Figure S1. Scheme of the transfer process of the GNW films on PE substrates. Figure S2. Flexible electrodes based on GNW films transferred on PE substrate (a—back view; b—front view). Figure S3. Flexible electrode based on GNW films transferred on PE substrate in a bent position (a—front view; b—back view; c—top view; d—side view; e—returning position to the original shape). Figure S4. Cyclic voltammetry (100 mV/s) illustrating the response of the GNW 15 min/PE electrode in the presence of 1 mM anthracene in an acetonitrile/water mixture (ACN/H_2_O, 80/20 *v*/*v*) containing 0.1 M LiClO_4_ as the supporting electrolyte. Figure S5. Repeatability tests of the GNW 45 min /PE electrode in the presence of 200 µM anthracene in an acetonitrile/water mixture (ACN/H_2_O, 80/20 *v*/*v*) containing 0.1 M LiClO_4_ as the supporting electrolyte; Table S1. Raman characteristics of the GNW films obtained at different growth times. Table S2. Heterogeneous electron transfer rate constants, *k*^o^, obtained using 2 mM K_3_[Fe(CN)_6_] dissolved in 10 mM phosphate-buffered saline solution (PBS) + 0.1 M KCl as the supporting electrolyte; cyclic voltammograms recorded in the 2–500 mV s^−1^ range. Table S3. Reported electrochemical sensors and methods for anthracene determination. Refs. [9,10,39,40,41,42,43,44,45] are cited in Supplementary Materials.
