# Supplementary material for: Vertically Aligned Nanocrystalline Graphite Nanowalls for Flexible Electrodes as Electrochemical Sensors for Anthracene Detection
Source: Sensors (Basel). 2024 Nov 10;24(22):7194. doi: 10.3390/s24227194 (PMC11598085; doi:10.3390/s24227194)
Supplement: Supplementary file 1 [file sensors-24-07194-s001.zip › sensors-3296134-supplementary.pdf]

Supplementary Materials

Vertically aligned nanocrystalline graphite nanowalls for flexible electrodes as electrochemical sensors for anthracene detection

Marius C. Stoian *, Octavian G. Simionescu, Cosmin Romanitan, Gabriel Craciun, Cristina Pachiu and Antonio Radoi *

National Institute for Research and Development in Microtechnologies—IMT Bucharest, 126A Erou Iancu Nicolae Street, 077190 Voluntari, Romania; octavian.simionescu@imt.ro (O.G.S.); cosmin.romanitan@imt.ro (C.R.); cristina.pachiu@imt.ro (C.P.)

* Correspondence: marius.stoian@imt.ro (M.C.S.); antonio.radoi@imt.ro (A.R.)

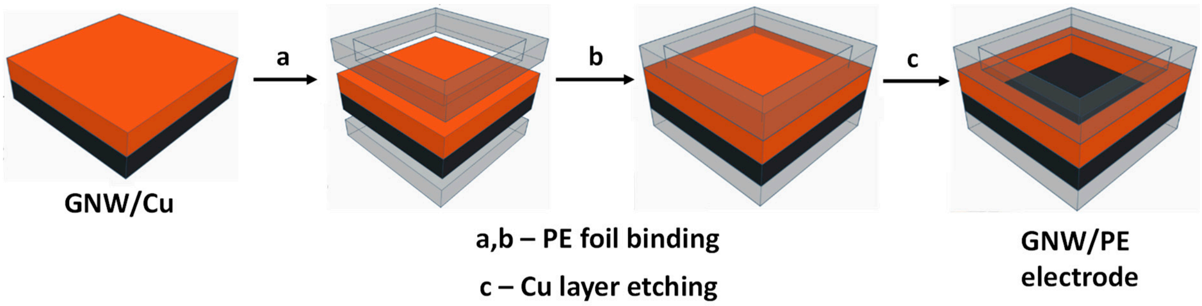

Figure S1. Scheme of the transfer process of the GNW films on PE substrates.

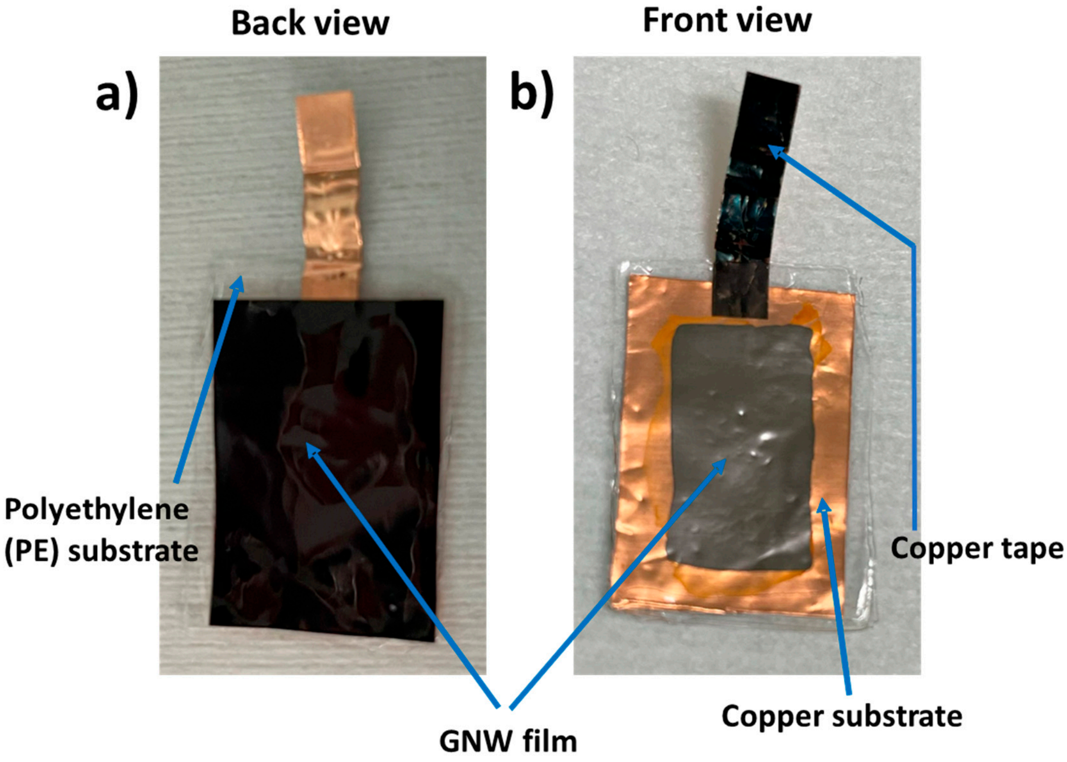

Figure S2. Flexible electrodes based on GNW films transferred on PE substrate (a – back view; b – front view).

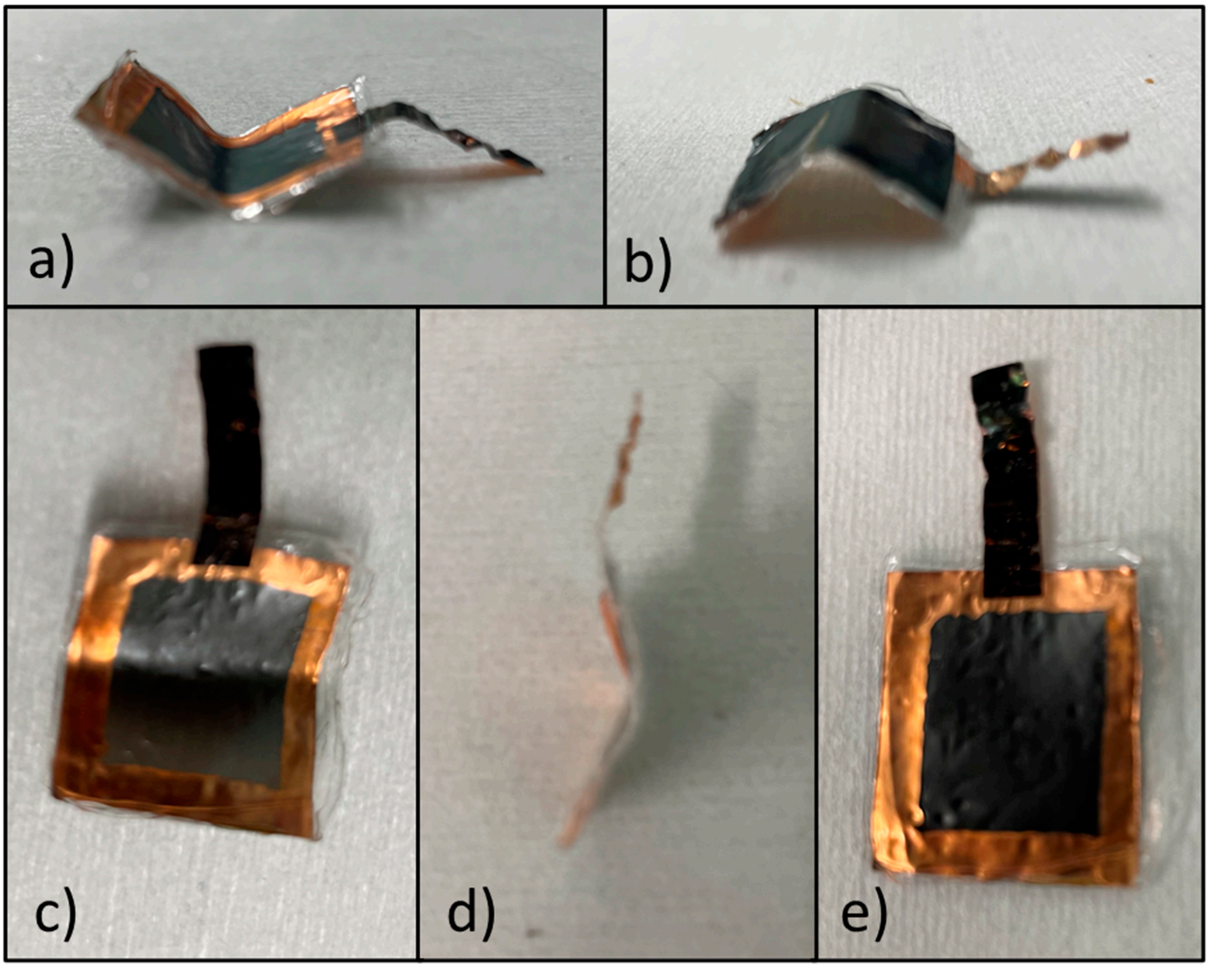

Figure S3. Flexible electrode based on GNW films transferred on PE substrate in a bent position (a – front view; b – back view; c – top view; d – side view; e – returning position to the original shape).

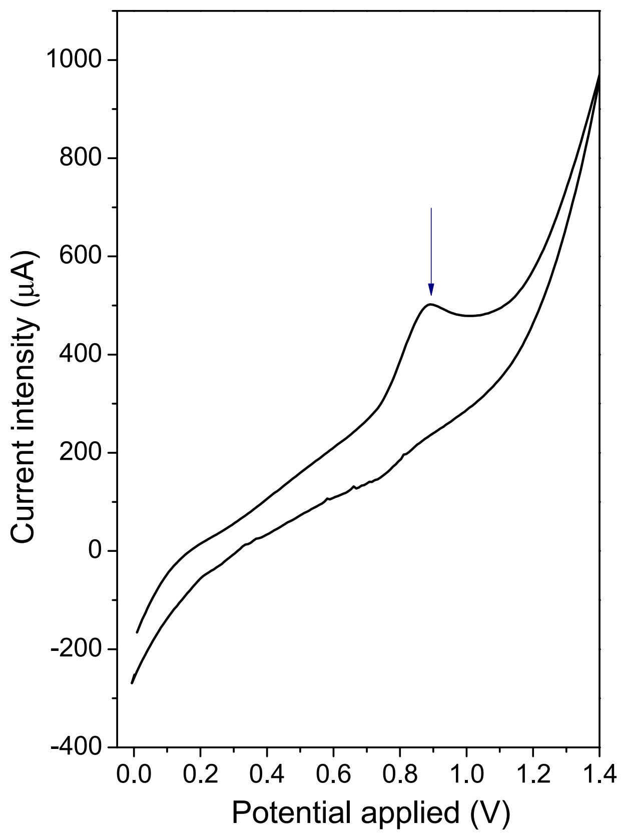

Figure S4. Cyclic voltammetry (100 mV/s) illustrating the response of the GNW 15 min/PE electrode in the presence of 1 mM anthracene in an acetonitrile/water mixture (ACN/H_2O, 80/20 v/v) containing 0.1 M $LiClO_4$ as the supporting electrolyte.

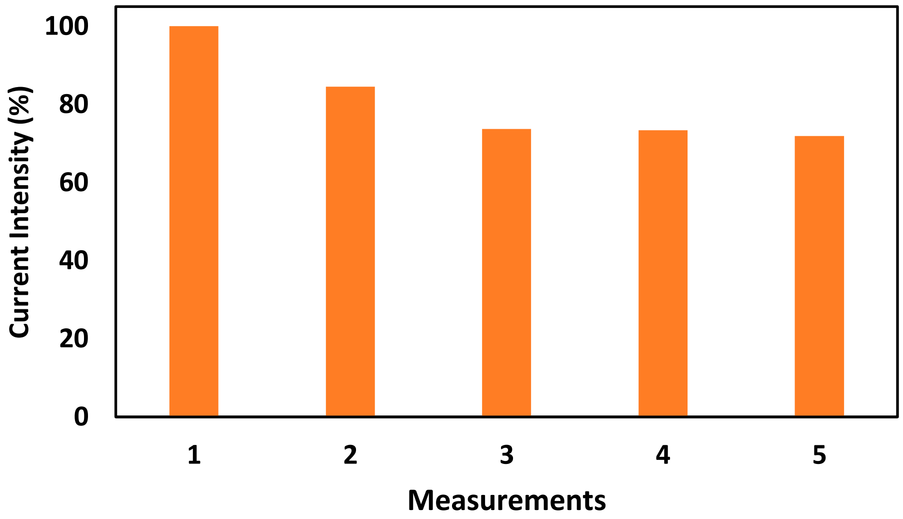

Figure S5. Repeatability tests of the GNW 45 min/PE electrode in the presence of 200 µM anthracene in an acetonitrile/water mixture (ACN/H_2O, 80/20 v/v) containing 0.1 M $LiClO_4$ as the supporting electrolyte.

Table S1. Raman characteristics of the GNW films obtained at different growth times.

Raman features/ Sample	GNW 15 min	GNW 30 min	GNW 45 min
D (cm^{-1})	1344	1349	1349
G (cm^{-1})	1579	1588	1588
2D (cm^{-1})	2691	2683	2685
D+D' (cm^{-1})	2931	2939	2937
I(D) (a.u.)	66043	51748	54004
I(G) (a.u.)	24659	21188	21618
I(D)/I(G)	2.68	2.44	2.50

Table S2. Heterogeneous electron transfer rate constants, *k*°, obtained using 2 mM $K_3[Fe(CN)_6]$ dissolved in 10 mM phosphate buffer saline solution (PBS) + 0.1 M KCl as supporting electrolyte, and cyclic voltammograms were recorded in the 2 - 500 mV s^{-1} range.

v (mV s^{-1})	GNW 15 min				GNW 30 min				GNW 45 min			
	ΔE (mV)	k° (cm s^{-1})	I_p (A)	k° (cm s^{-1}), average	ΔE (mV)	k° (cm s^{-1})	I_p (A)	k° (cm s^{-1}), average	ΔE (mV)	k° (cm s^{-1})	I_p (A)	k° (cm s^{-1}), average
2	73.24	2.59×10^{-3}	2.71×10^{-5}	**2.86 x 10^{-3}**	70.8	3.15×10^{-3}	2.35×10^{-5}	**5.24 x 10^{-3}**	70.8	3.15×10^{-3}	2.91×10^{-5}	**7.16 x 10^{-3}**
5	78.12	2.99×10^{-3}	4.39×10^{-5}		70.8	4.98×10^{-3}	3.76×10^{-5}		73.24	4.09×10^{-3}	4.42×10^{-5}	
10	85.45	2.97×10^{-3}	6.19×10^{-5}		73.24	5.79×10^{-3}	5.29×10^{-5}		73.24	5.79×10^{-3}	6.32×10^{-5}	
15	85.45	3.63×10^{-3}	7.57×10^{-5}		75.68	6.00×10^{-3}	6.54×10^{-5}		73.24	7.09×10^{-3}	7.65×10^{-5}	
20	85.45	4.19×10^{-3}	8.72×10^{-5}		75.68	6.93×10^{-3}	7.57×10^{-5}		75.68	6.93×10^{-3}	8.86×10^{-5}	
25	87.89	4.65×10^{-3}	1.06×10^{-4}		80.56	6.44×10^{-3}	9.32×10^{-5}		75.68	8.49×10^{-3}	1.09×10^{-4}	
30	90.33	4.90×10^{-3}	1.21×10^{-4}		85.45	5.93×10^{-3}	1.08×10^{-4}		75.68	9.80×10^{-3}	1.26×10^{-4}	
40	87.89	6.01×10^{-3}	1.35×10^{-4}		87.89	6.01×10^{-3}	1.20×10^{-4}		78.13	9.46×10^{-3}	1.41×10^{-4}	
50	139.16	2.02×10^{-3}	1.45×10^{-4}		92.78	6.15×10^{-3}	1.38×10^{-4}		85.45	8.12×10^{-3}	1.69×10^{-4}	

100	146.48	2.04×10^{-3}	1.65×10^{-4}		100.1	5.64×10^{-3}	1.61×10^{-4}		87.89	8.49×10^{-3}	1.96×10^{-4}	
150	163.58	1.85×10^{-3}	1.94×10^{-4}		104.98	6.02×10^{-3}	1.97×10^{-4}		92.77	8.70×10^{-3}	2.39×10^{-4}	
200	175.78	1.74×10^{-3}	2.16×10^{-4}		119.62	4.88×10^{-3}	2.25×10^{-4}		102.54	7.43×10^{-3}	2.72×10^{-4}	
250	183.1	1.73×10^{-3}	2.36×10^{-4}		126.96	4.67×10^{-3}	2.50×10^{-4}		107.42	7.29×10^{-3}	3.10×10^{-4}	
300	192.88	1.61×10^{-3}	2.54×10^{-4}		134.28	4.42×10^{-3}	2.72×10^{-4}		109.86	7.51×10^{-3}	3.33×10^{-4}	
350	200.2	1.53×10^{-3}	2.70×10^{-4}		136.72	4.56×10^{-3}	3.01×10^{-4}		117.19	6.82×10^{-3}	3.59×10^{-4}	
400	209.96	1.38×10^{-3}	2.82×10^{-4}		141.6	4.45×10^{-3}	3.21×10^{-4}		122.07	6.55×10^{-3}	3.82×10^{-4}	
450	214.84	1.34×10^{-3}	3.00×10^{-4}		146.48	4.32×10^{-3}	3.37×10^{-4}		124.51	6.59×10^{-3}	4.03×10^{-4}	
500	217.28	1.35×10^{-3}	3.11×10^{-4}		153.8	4.00×10^{-3}	3.11×10^{-4}		126.95	6.61×10^{-3}	4.24×10^{-4}	

Table S3. Reported electrochemical sensors and methods for anthracene determination.

Electrode	Applied potential / Electro-analytical method	Sensitivity (µA/µM)	LOD (M)	Dynamic range, µM	Ref.
Molecularly imprinted polypyrrole modified glassy carbon electrode (GCE)	1.06 V / SWV in 0.1 M H_2SO_4	1.23	1.2×10^{-8}	0.01– 0.375	[1]
Polyamic acid-graphene oxide nanocomposite screen printed carbon electrode (PPA-GO/SPCE)	-0.28 V / SWV in 0.1 HCl	9.86	6.7×10^{-7}	0.375 – 1.25	[2]
PPyox/Ag-AuNPs/GCE	1.18 V / SWV in 0.1 M $LiClO_4/CH_3CN$	0.141	1.69×10^{-7}	3 – 356	[3]
Graphenated Polyaniline/GCE	1.10 V / SWV in 0.1 M $LiClO_4/(CH_3CN/H_2O$, 80/20% v/v)	5.71×10^{-2}	4.4×10^{-9}	0.012 – 1000	[4]
Dendritic 7T-polythiophene/Au	1.51 V / CV in 0.1 M $LiClO_4/CH_3CN$	0.32	1.9×10^{-8}	0.04 – 0.2	[5]
MWCNTs/GCE	0.96 V / SWV in 0.1 M $LiClO_4/(CH_3CN/H_2O$, 80/20% v/v)	7.54×10^{-2}	4.2×10^{-5}	50 – 146	[6]
ZnO NPs/fMWCNTs/GCE	1.11 V / SWV in 0.1 M $LiClO_4/(CH_3CN/H_2O$, 80/20% v/v)	0.51	1.27×10^{-6}	9 – 75	[7]
N-NCG3/Si	0.69 V / DPV in 0.1 M $LiClO_4/(CH_3CN/H_2O$, 80/20% v/v)	0.75	5×10^{-6}	2.5 – 1000	[8]
GNW/PE	0.75 V / DPV in 0.1 M $LiClO_4/(CH_3CN/H_2O$, 80/20% v/v)	0.21	4.1×10^{-6}	2.5 – 100	[9]
GNW 45 min/PE	0.71 V / DPV in 0.1 M $LiClO_4/(CH_3CN/H_2O$, 80/20% v/v)	0.36	9.4×10^{-6}	1 – 500	This work

References SI:

1. Mathieu-Scheers, E.; Bouden, S.; Grillot, C.; Nicolle, J.; Warmont, F.; Bertagna, V.; Cagnon, B.; Vautrin-Ul, C. Trace Anthracene Electrochemical Detection Based on Electropolymerized-Molecularly Imprinted Polypyrrole Modified Glassy Carbon Electrode. *Journal of Electroanalytical Chemistry* **2019**, *848*, 113253, doi:10.1016/j.jelechem.2019.113253.

2. Hamnca, S.; Ward, M.; Ngema, X.T.; Iwuoha, E.I.; Baker, P.G.L. Development of Graphenated Polyamic Acid Sensors for Electroanalytical Detection of Anthracene. *Journal of Nano Research* **2016**, *43*, 11–22, doi:10.4028/www.scientific.net/JNanoR.43.11.

3. Mailu, S.N.; Waryo, T.T.; Ndangili, P.M.; Ngece, F.R.; Baleg, A.A.; Baker, P.G.; Iwuoha, E.I. Determination of Anthracene on Ag-Au Alloy Nanoparticles/Overoxidized-Polypyrrole Composite Modified Glassy Carbon Electrodes. *Sensors* **2010**, *10*, 9449–9465, doi:10.3390/s101009449.

4. Tovide, O.; Jahed, N.; Sunday, C.E.; Pokpas, K.; Ajayi, R.F.; Makelane, H.R.; Molapo, K.M.; John, S. V.; Baker, P.G.; Iwuoha, E.I. Electro-Oxidation of Anthracene on Polyanilino-Graphene Composite Electrode. *Sens Actuators B Chem* **2014**, *205*, 184–192, doi:10.1016/j.snb.2014.07.116.

5. Rassie, C.; Olowu, R.A.; Waryo, T.T.; Wilson, L.; Williams, A.; Baker, P.G.; Iwuoha, E.I. Dendritic 7T-Polythiophene Electro-Catalytic Sensor System for the Determination of Polycyclic Aromatic Hydrocarbons. *Int J Electrochem Sci* **2011**, *6*, 1949–1967, doi:10.1016/S1452-3981(23)18158-2.

6. Mwazighe, F.M. Multi-Walled Carbon Nanotubes-Modified Glassy Carbon Electrode for the Detection of Anthracene. *Int J Electrochem Sci* **2020**, *15*, 11058–11069, doi:10.20964/2020.11.64.

7. Adesanya, F.A.; Fayemi, O.E. Anthracene Electrochemical Sensor at FMWCNTs/ZnO Modified Glassy Carbon Electrode. *Int J Electrochem Sci* **2023**, *18*, 100382, doi:10.1016/j.ijoes.2023.100382.

8. Simionescu, O.-G.; Romanitan, C.; Albu, C.; Pachiu, C.; Vasile, E.; Djourelov, N.; Tutunaru, O.; Stoian, M.C.; Kusko, M.; Radoi, A. Properties of Nitrogen-Doped Nano-Crystalline Graphite Thin Films and Their Application as Electrochemical Sensors. *J Electrochem Soc* **2020**, *167*, 126510, doi:10.1149/1945-7111/abb1d4.

9. Stoian, M.C.; Romanitan, C.; Simionescu, O.G.; Djourelov, N.; Brincoveanu, O.; Dinescu, A.; Radoi, A. Growth of Nanocrystalline Graphite and Vertically Aligned Graphite Nanowalls Thin Films and Their Transfer on Flexible Substrates for Applications as Electrochemical Sensors for Anthracene Detection. *Microchemical Journal* **2024**, *207*, 111828, doi:10.1016/j.microc.2024.111828.
